# Prediction of tractor drawbar pull under different tillage tools using machine learning and low-cost sensors

**DOI:** 10.1038/s41598-025-24974-w

**Published:** 2025-11-20

**Authors:** So-Yun Gong, Si-Eon Lee, Yi-Seo Min, Seung-Min Baek, Seung-Yun Baek, Yong-Joo Kim, Wan-Soo Kim

**Affiliations:** 1 Department of Bio-Industrial Machinery Engineering, Kyungpook National University, Daegu, 41566 Republic of Korea; 2 Eco-Friendly Hydrogen Electric Tractor & Agricultural Machinery Institute, Chungnam National University, Daejeon, 34134 Republic of Korea; 3 Department of Agricultural and Biosystems Engineering, North Dakota State University, Fargo, ND 58102 USA; 4 Department of Smart Agricultural System Machinery Engineering, Chungnam National University, Daejeon, 34134 Republic of Korea; 5 Department of Smart Bio-Industrial Mechanical Engineering, Kyungpook National University, Daegu, 41566 Republic of Korea; 6 Upland Field Machinery Research Center, Kyungpook National University, Daegu, 41566 Republic of Korea

**Keywords:** Engineering, Mathematics and computing

## Abstract

Machine-learning models were developed to predict the drawbar pull of a 78-kW-class tractor for moldboard, chisel, and subsoiler plows. Four models were tested: random forest (RF), extreme gradient boosting (XGB), artificial neural network (ANN), and support vector machine (SVM). The training variables included engine speed (ES), engine torque (ET), travel speed (TS), tillage depth (TD), and slip ratio (SR). Unlike prior studies that focused mainly on engine parameters, this study incorporated nonlinear variables to improve both accuracy and practical applicability. Data were collected from three Korean paddy fields with different soil conditions, and the dataset was divided into 70% for training and 30% for testing. Five input variable combinations were used: Model A (ES, ET), Model B (ES, ET, TD), Model C (ES, ET, TS, SR), Model D (TD, TS), and Model E (ES, TD, TS). The results showed that, for the moldboard plow, RF in Model E achieved the highest performance (R^2^ = 0.977). For the chisel plow, ANN in Models B and C provided strong predictive accuracy (R^2^ = 0.953). The subsoiler also performed well with ANN in Models B and E (R^2^ = 0.953). Overall, the proposed models—particularly RF and ANN—proved effective in predicting drawbar pull and outperformed XGB and SVM. This study is distinguished by its comparison of various input variable combinations for different plows (moldboard, chisel, and subsoiler) and by its proposal of a cost-effective approach using low-cost sensors.

## Introduction

The global agricultural-machinery market is projected to grow from $155.1 billion in 2021 to $212.6 billion in 2026, with a compound annual growth rate (CAGR) of 6.5%. The tractor market alone is estimated to grow from $55.5 billion in 2021 to $77.8 billion in 2026 at a CAGR of 7.0%^1^. Tractors are widely used in agricultural fields owing to their versatility in plowing, mowing, harvesting, and transportation operations^2^. Tractors have the advantages of a wide transmission range and adaptability to diverse road conditions. A tractor performs agricultural tasks by attaching implements to its front or rear^3^ and primarily executes agricultural tasks by towing attached implements or supplying power through a power take-off (PTO). Tractors are typically operated on soil, which has diverse and irregular characteristics owing to varying physical and chemical properties^4^.

The plowing operation of tractors requires a substantial drawbar pull owing to high soil resistance; consequently, the traction performance of a tractor is a critical aspect of its design. The evaluation of the traction performance of a tractor is currently based on the Organization for Economic Co-operation and Development (OECD) testing method, which involves towing a drawbar equipped with various sensors on asphalt road surfaces^5^. Although evaluation under concrete-road conditions is useful for comparing performances across various models and manufacturers, it fails to reflect actual soil conditions, resulting in significant discrepancies with agricultural performance^6^. Tractor power-transmission efficiency, traction efficiency, and work performance are significantly influenced by losses caused by slip and other factors resulting from the complex interactions between tractor driving wheels and the soil^7^. Numerous studies have used load and traction measurement systems to evaluate the traction performance of tractors. Battiato and Diserens (2017) conducted simulations and experimental validations of tractor traction performance across various soil textures to enhance the practical applicability in agricultural operations^8^. Their analysis considered factors such as soil–tire interaction, tractor configuration, slip ratio, and tire pressure. Kumari and Raheman (2019) developed an embedded advisory system to evaluate the traction performance of tractors by optimizing the gear and throttle settings^9^. The system measures the wheel slip, velocity ratio, and engine load in real time, providing operators with guidance to maintain optimal ranges, thereby improving both traction performance and fuel efficiency. However, evaluating tractor traction performance remains cumbersome because it requires complex measurement systems using various sensors, followed by data collection and analysis through experimentation^10^. Thus, a technology that can predict tractor traction performance based on key tractor component data is required.

Various attempts have been made to predict traction performance. Before the introduction of machine learning, most studies employed statistical approaches with widely used regression models. Askari et al. (2021) utilized a response surface methodology approach to predict the tractive performance of agricultural tractors^11^. Ranjbarian et al. (2017) used the American Society of Agricultural and Biological Engineers (ASABE) equations to predict the tractor slip rate, drawbar pull, and traction efficiency. However, with the rapid advancement of artificial intelligence technologies and high-performance computing techniques, machine-learning methods are increasingly being applied in various areas of agricultural machinery for performance prediction^12^.

Factors influencing tractor traction performance include soil properties, soil-tractor interactions, and the type of attached implement^13^. Of these, the type of attached implement plays a critical role in determining the traction performance. In particular, plows, commonly used for tillage, significantly affect tractor traction performance, with variations depending on the type of plow^14^. For example, the type of plow influences factors such as the tractor speed and tillage depth, which ultimately have a direct impact on traction performance. Although studies have been conducted on the traction performance of tractors equipped with various plows, further research is required to address the remaining gaps. Arvidsson et al. (2004) measured the specific drawbar pull and energy consumption of three plowing tools (moldboard plow, chisel plow, and disc plow) under various soil conditions and demonstrated that the required drawbar pull varied depending on the plow type under identical soil conditions^15^. Kim et al. (2022) analyzed the effects of tillage depth and soil properties on the working performance of a tractor-moldboard plow system and confirmed that the engine load and drawbar pull varied considerably depending on the soil type and tillage depth^16^. Upadhyay et al. (2024) employed ANN and regression models to predict the power requirements of agricultural machinery and applied the ANN–PSO (Particle Swarm Optimization) technique to determine the optimal operating conditions^17^. Nataraj et al. (2021) developed ANN and regression models using soil properties and operating conditions as input variables to predict the draft and power requirements of a rotary tiller, and compared their performance^18^. Upadhyay et al. (2025) developed a regression model to predict the draft and torque requirements of an active–passive disc harrow (APDH) and derived the optimal operating conditions through multi-objective optimization based on a genetic algorithm^19^. These studies concluded that the type of plow significantly affects traction performance. Furthermore, studies have been conducted to predict the tractor performance under specific working conditions. However, previous studies mainly employed expensive high-cost sensors, did not sufficiently consider diverse input variables, and mainly focused on evaluating tractor traction performance with a single type of plow. Considering different plows exhibit distinct performance characteristics in terms of the drawbar pull, slip ratio, and soil disturbance, a comprehensive approach that accounts for these variations is required; research comparing and predicting the traction performance of tractors equipped with multiple types of plows under various field conditions remains insufficient.

This study aims to analyze and predict the traction performance of tractors equipped with different types of plows, focusing on their impact on key traction parameters. The specific objectives of this study were as follows: (1) Evaluate the traction performance of tractors equipped with three distinct types of plows (moldboard, chisel, and subsoiler) under varying soil conditions, using a load-measurement system in field experiments. (2) Assess the impact on the overall drawbar pull by systematically measuring key traction-related variables, including the engine load (torque, speed, and power), travel speed, slip ratio, tillage depth. (3) Develop and validate a predictive model that predicts the tractor traction performance for different plow types based on machine-learning techniques, incorporating the real-world operational data collected from field experiments.

## Methods

### Measurement system

In this study, the performance of a 78-kW tractor (S07, TYM Co., Ltd., Korea) was measured. The dimensions of the tractor are 4225 mm (length) × 2140 mm (width) × 2,830 mm (height), with a weight of 3985 kg. The rated engine power is 78 kW (at 2300 rpm), and the maximum torque is 430 Nm (at 1400 rpm). The transmission system provides 32 forward and 32 reverse gear stages, achieved through a four-speed main transmission, a four-range transmission, and a two-speed power-shift function for high- and low-range switching. In addition, a four-stage sub-transmission is available, providing a variety of gear-selection options. The maximum recorded drawbar pull was 29.25 kN, with a traction power of 11.55 kW, achieved at a speed of 1.41 km/h. When operating in the gear closest to 7.5 km/h, the traction performance was measured at a traction power of 48.23 kW, a drawbar pull of 25.74 kN, and a travel speed of 6.75 km/h. The maximum recorded traction power was 56.59 kW, with a drawbar pull of 16.68 kN, at a speed of 12.23 km/h.

**Fig. 1:**
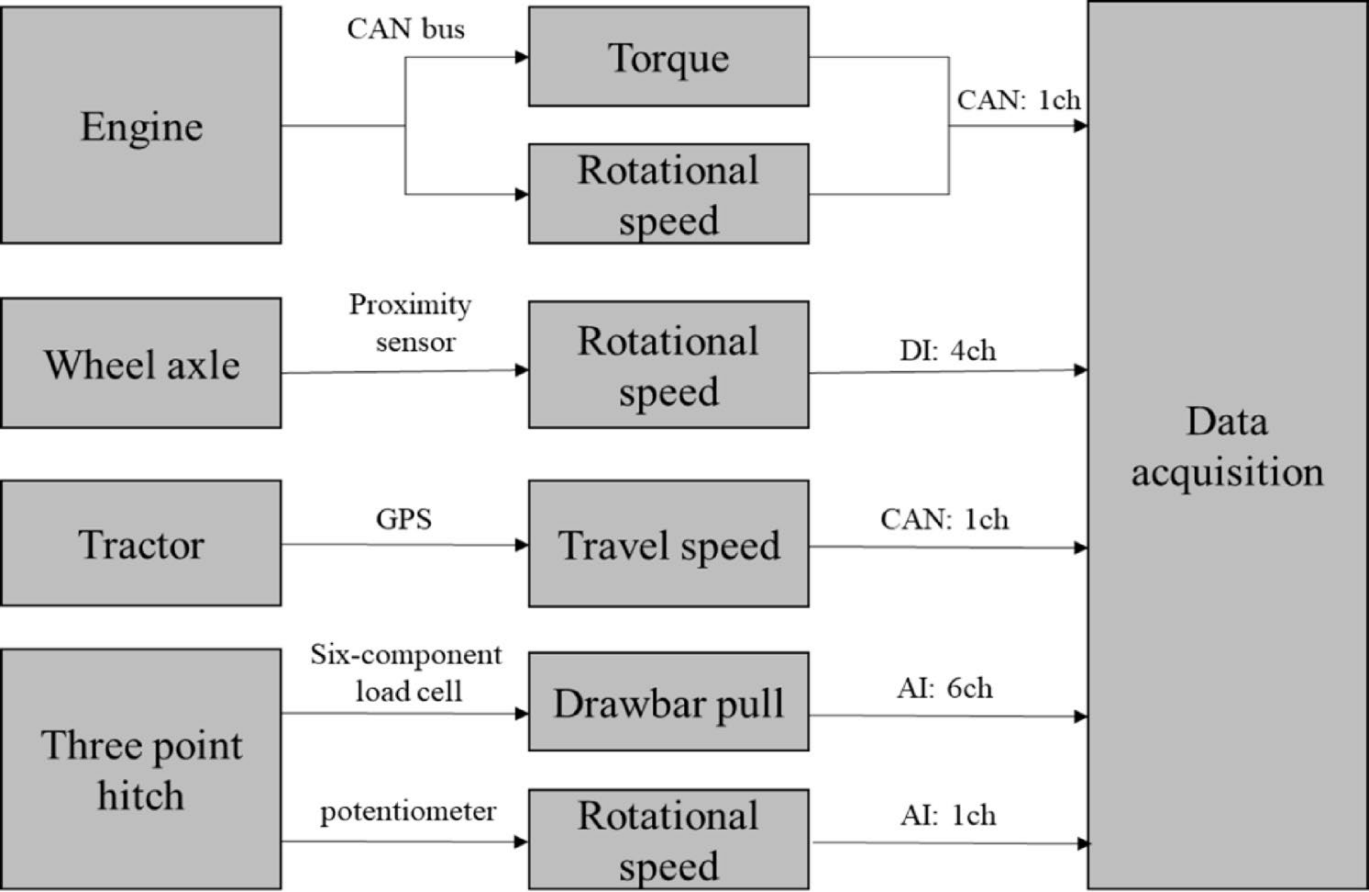
Diagram of the tractor field data acquisition system.

A measurement system was designed to measure the engine, axle, drawbar, and other key operating variables of a tractor in real-time, as shown in Fig. 1. The engine torque and rotational speed were measured via controller-area-network (CAN) communication and transmitted to the data-acquisition device through CAN channels. The rotational speed was measured using a proximity sensor (PRDCMT30-25DO; Autonics, Korea). The torque and rotational speed of the wheel axles were measured using analog input (four channels) and digital input (four channels). A GPS device (GPS 18x, Garmin, USA) was mounted on top of the tractor cabin to measure the driving speed, and the data were transmitted to the data-acquisition device through CAN channels. The plowing depth was measured by attaching a potentiometer to the three-point hitch, and data were collected through an analog input channel (1 channel). All collected data were stored in real time using a DAQ system (CRONOS Compact CRC-400-11, IMC, Germany).To measure the drawbar pull, a six-axis force meter was attached between the tractor and rear-mounted implement. The six-axis force meter consisted of six load cells (DACELL, UU-T2, Korea), and equations were used to calculate the drawbar pull using the data collected by three of these load cells.

Table 1 presents the measurement accuracy and error specifications of the sensors used in this study. The DACELL UU-T2 showed an accuracy within ±0.1% R.O., while the potentiometer exhibited a linearity of ±1% and an end-point sensitivity of ±1% of the supply voltage. The Autonics PRDCMT30-25DO had a response frequency of up to 100 Hz with a detection distance tolerance within ±10%, and the Garmin GPS 18x provided a position accuracy of less than 3 m and a velocity error of less than 0.1 m/s.

**Table 1:** Specifications of measurement accuracy and error for the sensors used in this study.

Sensor	Measurement item	Accuracy / Error specification
DACELL UU-T2	Drawbar pull	Nonlinearity, hysteresis, and repeatability within ± 0.1% R.O
Potentiometer	Tillage depth	Linearity: ± 1% Sensitivity of the end points: ± 1% of supply voltage
Autonics PRDCMT30-25DO	Rotational speed	Response frequency up to 100 Hz, detection distance tolerance within ± 10%
Garmin GPS 18x	Travel speed	Position accuracy < 3 m (WAAS enabled), velocity error typically < 0.1 m/s

### Field experiment

**Fig. 2:**
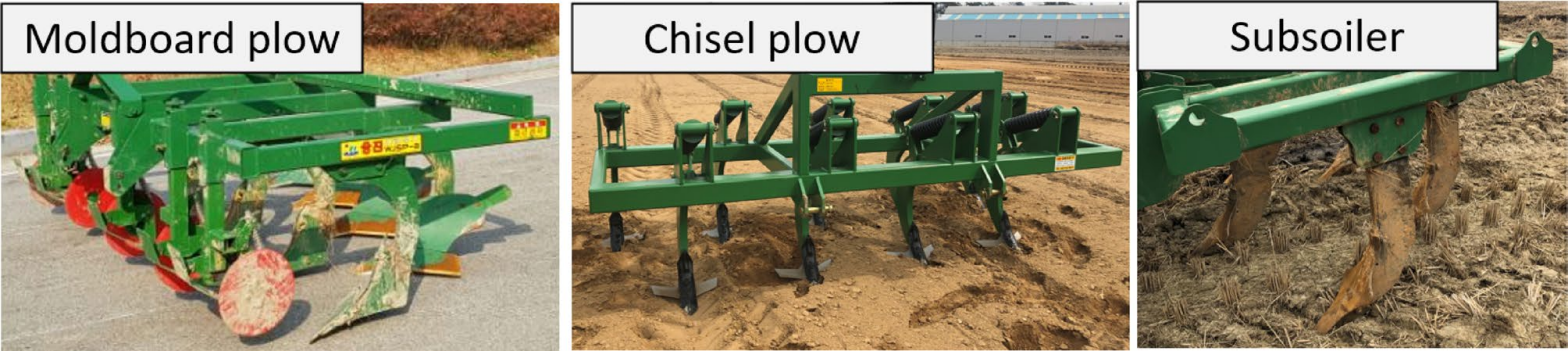
Tillage implements used in this study.

*Types of agricultural implements* Various types of plows, such as moldboards, chisels, and subsoiler, are commonly used in agriculture^20^. Three types of implements were used in this study, as shown in Fig. 2. These include the moldboard plow, which is used to effectively cut soil and create furrows^21^ the chisel plow, which tills deep soil with its long, sharp blades^22^ and the subsoiler, which breaks through compacted hardpan layers^23^.

*Specifications of plows* The working widths of the moldboard and chisel plow were 2.8 m, whereas that of the subsoiler was 2.1 m. The maximum tillage depths are 0.2 m, 0.4 m, and 0.7 m for the moldboard plow, chisel plow, and subsoiler, respectively.

**Fig. 3:**
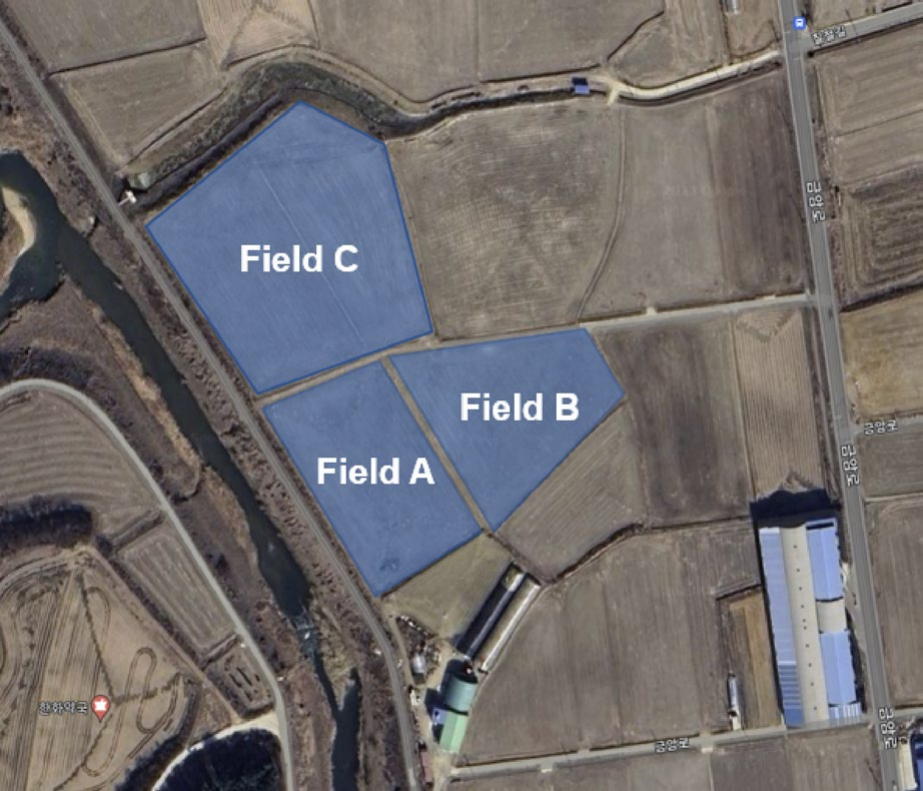
Field experiment sites for tractor-performance measurement.

*Site selection* Field experiments were conducted in rice paddy fields located in Geumam-ri, Songsan-myeon, Dangjin-si, Chungcheongnam-do, as shown in Fig.  3. Field A, measuring 6000 m^2^ (60 × 100 m), was used for the moldboard-plow operation. Field B, measuring 9800 m^2^ (120 × 80 m), and Field C, measuring 12,000 m^2^ (120 × 100 m), were used for both chisel-plow and subsoiler operations.

*Soil-environment analysis* The cone index was measured using a soil penetrometer (SC 900, Spectrum Technologies, Aurora, IL, USA) in accordance with ASABE standards^24^, and the cone index for each depth was presented as the average value for the 0–150 mm range. Soil-moisture content (SMC) was measured using a soil-moisture sensor (TDR 350, Spectrum Technologies, Aurora, IL, USA). Soil samples were collected from 10 uniform locations at each site, based on the plowing depth. Soil texture was analyzed based on the particle distribution of sand, silt, and clay as defined by the USDA^25^. The soil properties were analyzed for each field: the soils in Fields A, B, and C were loam, with similar results observed for both soil strength and moisture (Table 2).

**Table 2:** Soil environment analysis results by field experiment site.

Site	Cone index (kPa) for depths (0–15 cm)	Soil moisture content (%)	Soil texture
Field A	315–3684	37.63	Loam
Field B	270–3743	32.30	Loam
Field C	209–3100	35.77	Loam

**Fig. 4:**
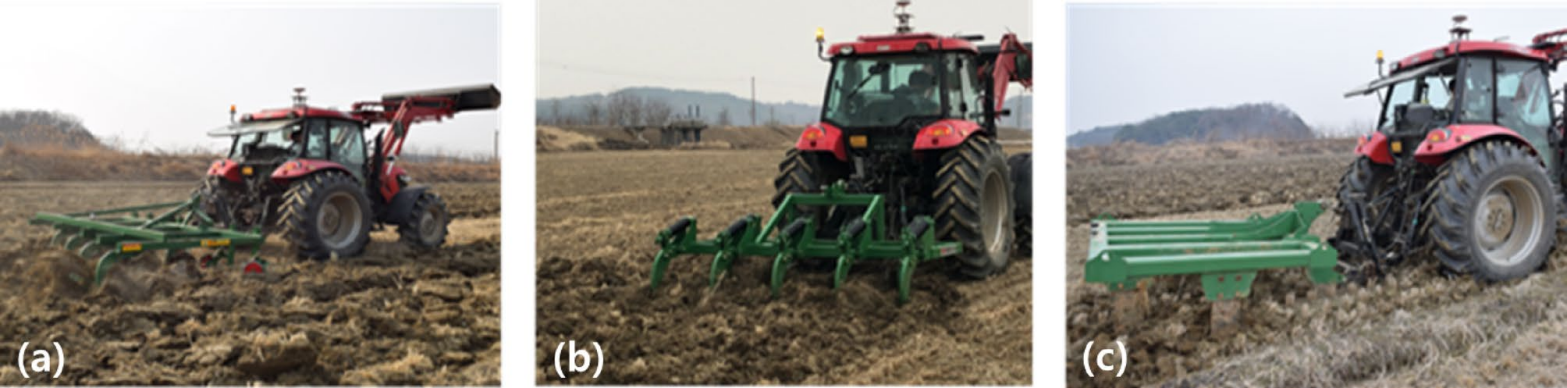
Field experiments with different implements: (**a**) moldboard plow, (**b**) chisel plow, and (**c**) subsoiler.

*Plowing-depth and gear settings* Field experiments were conducted for data measurement, as shown in Fig.  4. The plowing depth was set to 11–18 cm for the moldboard plow, 21–30 cm for the chisel plow, and 36–45 cm for the subsoiler. The operating gear was set to low speed M3 (7.1 km/h) for the moldboard plow and chisel plow, and low-speed L3 (2.4 km/h) for the subsoiler.

### Data analysis

The engine torque measured and rotational speed were used to calculate engine power required (Eq. (1)).

1 $$P_{a} = \frac{2\pi TN}{{60,000}}$$

where ${P}_{a}$ is the engine-power requirement (kW), T is the torque (Nm), and N is the engine rotational speed (rpm).

The drawbar pull was calculated by summing the tensile forces from the two lower load cells and the compressive force from the upper load cell^26^ (Eq. (2)).

2 $$F = F_{TC} + F_{LL} + F_{LR}$$

where F is the drawbar pull (kN), ${F}_{TC}$ is the compressive force on the top link (kN), ${F}_{LL}$ is the tensile force on the lower-left link (kN), and ${F}_{LR}$ is the tensile force on the lower-right link (kN).

The traction-force equation presented by the ASAE is shown in Eq. (3)^20^. Using this equation, the theoretical drawbar pull was calculated and compared with the drawbar pull obtained from experiments.

3 $$D = F_{i} \left[ {A + B\left( S \right) + C\left( S \right)^{2} } \right]WT$$

where D is the draft force from ASABE (kN), ${F}_{i}$ denotes the soil-resistance factor, A, B, and C are empirical constants, S is the working speed (m/s), W is the width of the implement (m), and T is the tillage depth (m).

Table 3 presents the predicted draft force for the various tillage and seeding implements using the model parameters. This table provides the parameter values used in the calculation for each implement based on the draft force equation presented in the ASAE standard D497.4^20^. A, B, and C are the coefficients that reflect the characteristics of each implement and play a critical role in predicting the draft force. Fi represents the drawbar pull and W denotes the width of the implement. For the medium-textured soils, the value of i = 2, specifically, F_2_, was used.

**Table 3:** Key parameters for estimating draft force of different tillage implements.

Item	A	B	C	***F***_***i***_ (F_2_)	W (m)
Moldboard plow	652	0	5.1	0.7	2.8
Chisel plow	123	7.3	0	0.85	2.8
Subsoiler	294	0	2.4	0.7	2.095

The coefficient of variation (CV) was calculated using Equation (4) to analyze the relative variability of the data. To examine the effects of different plow conditions on tractor performance, one-way analysis of variance (ANOVA) and post-hoc analysis using the least significant difference (LSD) method were conducted using IBM SPSS Statistics (SPSS 25, SPSS Inc., New York, USA). A statistically significant difference was determined at *p* < 0.05, which was satisfied by comparisons between multiple groups.

4 $$CV = \frac{SD}{{Average}}$$

where CV denotes the coefficient of variation and SD is the standard deviation.

### Machine learning models for predicting drawbar pull

**Fig. 5:**
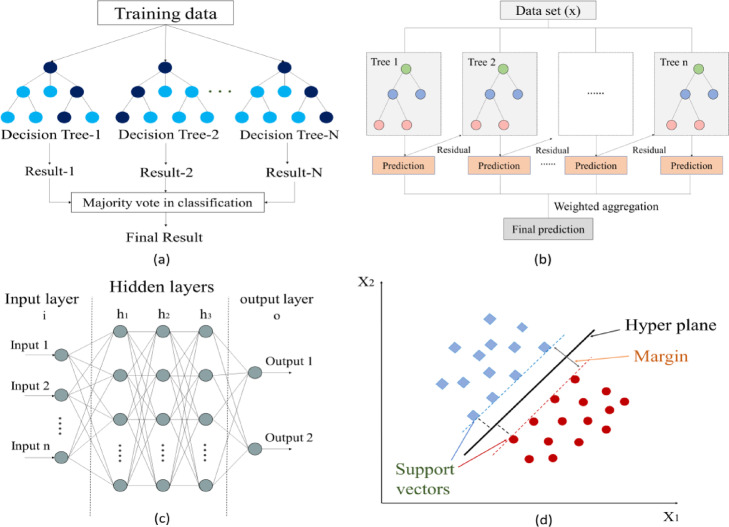
Overview of machine-learning models: (**a**) Random Forest (RF)—ensemble of decision trees with majority voting, (**b**) Extreme Gradient Boosting (XGB)—sequential boosting process, (**c**) Artificial Neural Network (ANN)—multi-layer perceptron with hidden layers, and (**d**) Support Vector Machine (SVM)—optimal hyperplane with support vectors.

The machine learning techniques used in this study include random forest (RF), extreme gradient boosting (XGB), artificial neural networks (ANN), and support vector machines (SVM). A structural overview of each model is presented in Fig. 5. Of the measured data, 70% were allocated to the training set, whereas the remaining 30% were used for the testing set.

Table 4 presents the designs of the five models for predicting the drawbar pull by combining different input variables. Scenario A refers to a situation in which the drawbar pull can be predicted using only engine data. When using a tractor equipped with an electronically controlled engine that supports CAN communication, engine data can be collected without the need for additional sensors^27^. Scenario B represents a situation in which the tillage depth is used as an additional input variable to improve the accuracy of the drawbar pull prediction. As the tillage depth is an important factor affecting the drawbar pull, incorporating it into the prediction model can enhance the performance^28^. Scenario C describes a case in which a speed sensor is used to measure the driving speed for drawbar pull prediction. The prediction performance can be improved by measuring the driving speed and using it to calculate the slip rate^29^. Scenario D refers to a situation in which the drawbar pull is predicted using only the tillage depth and travel speed without using engine data. In this case, the drawbar pull prediction was conducted without relying on engine-related data. Scenario E represents a situation in which the drawbar pull prediction is performed using all available data, including the engine, tillage depth, and speed sensor data. This approach assumes that increasing the number of input variables leads to improved prediction accuracy^30^.

**Table 4:** Combination of variables used for model development.

Model	A	B	C	D	E
Source	Engine speed + Engine torque	Engine speed + Engine torque + Tillage depth	Engine speed + Engine torque + Travel speed + Slip ratio	Tillage depth + Travel speed	Engine speed + Tillage depth + Travel speed

Table 5 compares the input variables, advantages, and disadvantages of different drawbar pull prediction methods. The conventional dynamometer-based approach ensures high accuracy. however, it is limited by the high cost of equipment. In contrast, ML-based models can effectively predict drawbar pull by integrating engine and soil variables that can be collected at relatively low cost.

**Table 5:** Comparison of measurement, input, strengths, and limitations of drawbar pull prediction methods.

Category	Measurement/Input	Strengths	Limitations
Conventional	Direct measurement with a dynamometer	Accurate calculation of drawbar pull	Requires additional equipment between tractor and implement, higher cost
Model A	Engine speed + Engine torque	Data acquisition from ECU without additional sensors	Low resolution and weak direct correlation with drawbar pull
Model B	Engine speed + Engine torque + Tillage depth	Considers soil resistance	In addition to engine-based data acquisition, the installation of additional sensors and data synchronization are required
Model C	Engine speed + Engine torque + Travel speed + Slip ratio	Reflects soil–tractor interaction by incorporating slip ratio	
Model D	Tillage depth + Travel speed	Reflects soil resistance and speed effect	
Model E	Engine speed + Tillage depth + Travel speed	Comprehensive reflection of engine, soil, and operating conditions with high accuracy	

Table 6 compares the costs of the low-cost sensors used in this study with those of commonly employed standard sensors. While the price of a conventional load cell amounts to approximately 5,500 USD, the total cost of the potentiometer, proximity sensor, and GPS receiver utilized in this study is only about 220 USD. This finding not only demonstrates that low-cost sensors can serve as a highly cost-effective alternative, but also indicates that a system capable of predicting drawbar pull can be constructed at roughly 4% of the cost of conventional systems. Furthermore, even when accounting for potential fluctuations in sensor prices, the system can still be implemented at less than 5% of the conventional cost.

**Table 6:** Comparison of low-cost sensors used in this study with commonly used standard sensors.

Items	Conventional sensor	Low-cost Sensor used in this study	
	Price (USD)	Model	Price (USD)
Drawbar pull sensor	5500	–	–
Potentiometer	–	Potentiometer	Approx. 80
Proximity sensor	–	Autonics PRDCMT30-25DO	Approx. 40
GPS	–	Garmin GPS 18x	Approx. 100
Total	5500	–	Approx. 220

The optimal hyperparameter values derived for each technique are listed in Table 7. In this study, the Optuna optimization method was used to determine the optimal hyperparameters. Specifically, a Bayesian optimization approach based on a tree-structured parzen estimator (TPE) was employed to efficiently search for optimal hyperparameters within the exploration space. The selection of hyperparameters varies by the model, making it essential to focus solely on the most significant hyperparameters. Removing unnecessary hyperparameters reduces computational complexity and shortens the training time, leading to more efficient resource utilization^31^.

**Table 7:** Hyperparameter combinations used to train a machine-learning model for predicting drawbar pull.

RF	XGB	ANN	SVM
n_estimators: 135 max_depth: 10 num_leaves: 43 learning rate: 0.09	n_estimators: 150 max_depth: 29 learning rate: 0.06 colsample_bytree: 0.82	n_layers: 4 units: 189 dropout_rate: 0.105 learning_rate: 0.001	c: 1.0 kernel: ‘rbf’ gamma: 0.02

*RF* The RF technique is a decision-tree-based ensemble learning algorithm that constructs multiple decision trees using bootstrap sampling and aggregates the results to develop a final predictive model. This ensemble method helps prevent overfitting by aggregating the predictions from multiple decision trees. The training process is controlled by hyperparameters such as the number of trees, maximum depth, and criteria for node splitting. Optimizing the hyperparameters is essential for developing an effective RF model, as it ensures high predictive performance while minimizing the risk of overfitting^32^. To optimize the model performance, the number of trees was set to 135, with a maximum depth of 10 and 43 leaf nodes, ensuring a balance between predictive accuracy and model complexity.

*XGB* The XGB is a gradient-boosting algorithm that sequentially trains weak learners by minimizing residual errors from previous iterations, thereby enhancing the predictive performance^33^. Unlike RF, XGB can be sensitive to the data scale because of its use of L1 and L2 regularization in the training process. Therefore, the traction data were normalized to a range of 0–1 before the model training. The number of trees was set to 150 and the maximum depth was adjusted to 29 to ensure sufficient model complexity. The learning rate was set to 0.06 to prevent overfitting while promoting fast convergence.

*ANN* An ANN is a computational model consisting of multiple neurons arranged in hidden layers, where the network adjusts weights during training to minimize prediction errors^34^. To optimize the network depth, four hidden layers were applied, with each hidden layer consisting of 189 neurons. In addition, a dropout rate of 0.105 was applied to improve the generalization performance of the network and prevent overfitting.

*SVM* The SVM is a supervised learning algorithm that determines the optimal hyperplane to maximize the margin between classes, making it effective for classification and regression tasks. In this study, the radial-basis-function kernel was applied with a regularization parameter (C) of 1.0 and a gamma value of 0.02, to balance the model complexity and generalization ability.

### Performance evaluation parameters

The metrics selected for evaluating the developed prediction models were the coefficient of determination (R^2^), root mean square error (RMSE), mean absolute percentage error (MAPE), and relative deviation (RD), as shown in Eq. (5–8). These metrics are widely used in regression analysis and model evaluation, making them suitable for assessing the predictive accuracy of the proposed models. R^2^ represents the proportion of variance in the dependent variable that is predictable from the independent variables, indicating how well the model captures variations in the actual data. RMSE is the square root of the mean of the squared differences between actual and predicted values, making it more sensitive to large errors than the mean absolute error (MAE), which simply averages absolute differences. The MAPE represents the mean of all absolute percentage errors normalized by the mean of the actual values, allowing a relative comparison of errors across different datasets. RD is a metric that normalizes the mean prediction error of the model using the mean measured value, providing a relative measure of model accuracy in predicting drawbar pull. A lower RD value indicates that the predicted values are closer to the actual values.

5 $$R^{2} = \frac{{\mathop \sum \nolimits_{i = 1}^{i = N} \left( {y_{i} - y_{a} } \right) - \mathop \sum \nolimits_{i = 1}^{i = N} \left( {y_{i} - \hat{y}_{i} } \right)}}{{\mathop \sum \nolimits_{i = 1}^{i = N} \left( {y_{i} - y_{a} } \right)}}$$

6 $$RMSE = \sqrt {\frac{1}{N}\mathop \sum \limits_{i = 1}^{i = N} \left( {\hat{y}_{i} - y_{i} } \right)^{2} }$$

7 $$MAPE = \frac{1}{N}\mathop \sum \limits_{i = 1}^{i = N} \left| {\frac{1}{{y_{i} }}\left( {y_{i} - \hat{y}_{i} } \right)} \right| \times 100\left( \% \right)$$

8 $$RD = \frac{RMSE}{{Mean }} \times 100\left( \% \right)$$

where ${y}_{a}$ is the mean measured drawbar pull, ${y}_{i}$ is the ith measured drawbar pull, and $\widehat{{y}_{i}}$ is the ith predicted drawbar pull.

Using the test data, the model was evaluated using the above evaluation metrics: R^2^, RMSE, MAPE, and RD. During the development of the prediction model, it was crucial to identify potential overfitting, which occurs when a model becomes overly tailored to the training data, leading to poor generalization. To assess overfitting, the test-to-train loss ratio (TTLR) (the ratio of MSE between the training and test models) was calculated; a TTLR < 1.5 indicates overfitting and a TTLR > 1.5 indicates underfitting. Furthermore, the R^2^ gap, which indicates the difference in the coefficient of determination between the training and test models, was used to evaluate the changes in the model accuracy on the test dataset. A large R^2^ gap indicates potential overfitting owing to better model performance on training than on the test data.

9 $$MSE = \frac{1}{N} \mathop \sum \limits_{i = 1}^{i = N} \left( {y_{i} - \hat{y}_{i} } \right)^{2}$$

10 $$TTLR = \frac{{MSE_{test} }}{{MSE_{train} }}$$

11 $$R^{2} Gap = \left| { R^{2}_{train} - R^{2}_{test} } \right|$$

where ${y}_{i}$ denotes the ith measured and $\widehat{{y}_{i}}$ denotes the ith predicted drawbar pulls, respectively.

### Robustness test

To validate the robustness of the developed model, additional data were collected and utilized. For this validation, robustness testing was performed only with the moldboard plow, as field operation data with other implements were not available from additional sites. This was because the moldboard plow is widely recognized as a standard implement for primary tillage, making it an appropriate reference tool to assess the generalizability of the proposed model. Specifically, Field D was located in Seosan-si, Chungcheongnam-do, and Field E in Anseong-si, Gyeonggi-do. Soil analysis revealed that Field D was classified as loamy sand, while Field E was classified as clay loam. Furthermore, the physical properties of the soil indicated that the cone index (0–15 cm depth) of Field D was 483 kPa with a soil moisture content of 33.79%, whereas Field E had a cone index of 1034 kPa and a soil moisture content of 33.60%^35^. These differences highlight the distinct soil conditions between the two test fields and served as an important basis for validating the robustness of the proposed model.

### Research framework and workflow

**Fig. 6:**
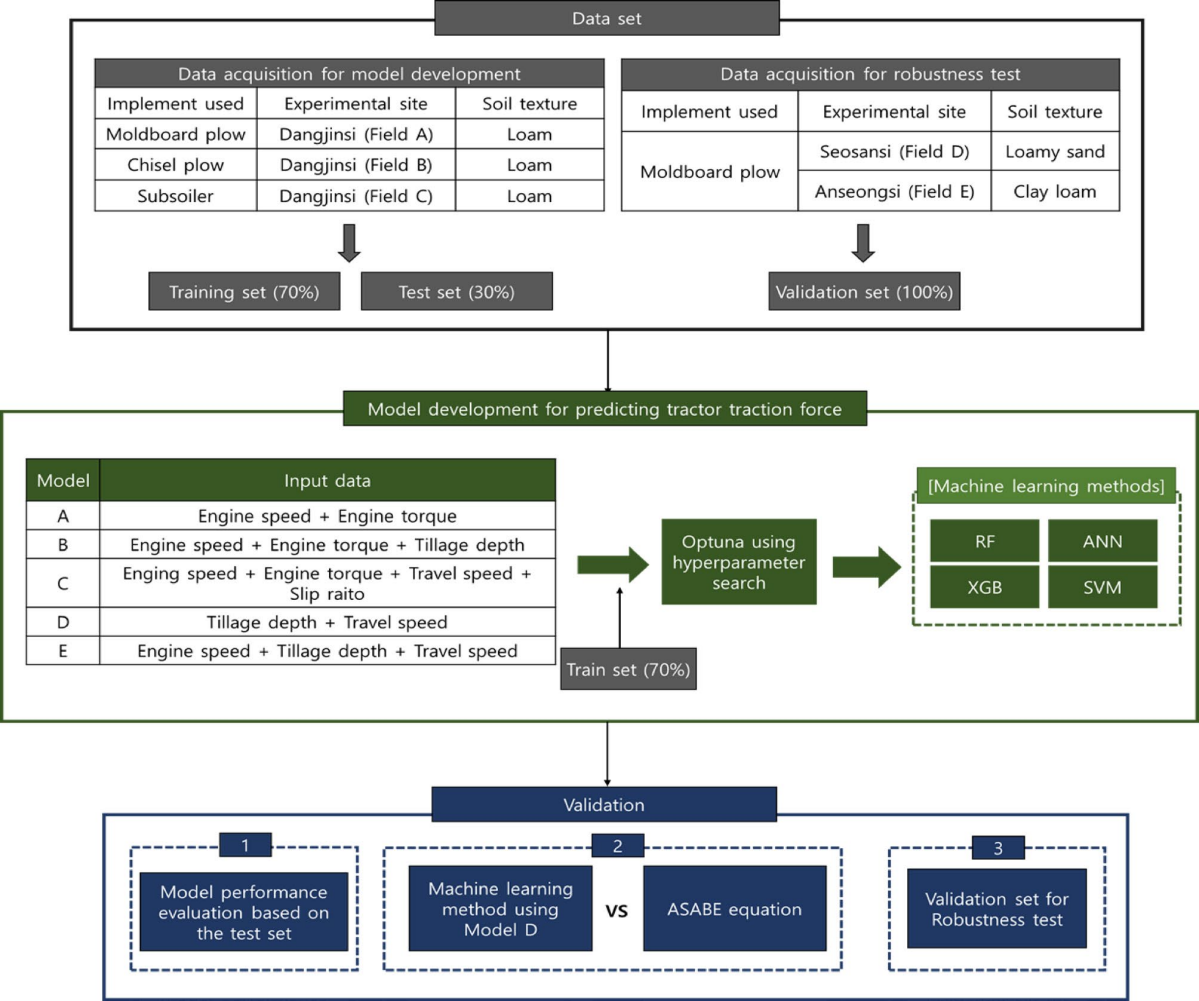
Framework for developing and validating machine learning-based drawbar pull prediction model.

Figure 6 provides a comprehensive overview of the research framework applied in this study, which consists of three main stages: data acquisition, model development, and validation. In the data acquisition stage, drawbar pull data were collected from three experimental fields in Dangjinsi, Chungcheongnam-do, using different implements: a moldboard plow (Field A), a chisel plow (Field B), and a subsoiler (Field C). All three sites were classified as loam. The dataset was divided into training (70%) and testing (30%) sets. In addition, for robustness validation, independent datasets were collected in Seosansi, Chungcheongnam-do (Field D, loamy sand) and Anseongsi, Gyeonggi-do (Field E, clay loam) using a moldboard plow. These datasets were used exclusively as the validation set. In the model development stage, five models (A–E) were constructed based on different combinations of input variables, including engine speed, engine torque, tillage depth, travel speed, and slip ratio. Hyperparameter optimization was performed using Optuna, and four machine learning methods—RF, ANN, XGB, and SVM—were applied. The validation stage consisted of three procedures. First, model performance was evaluated using the test set. Second, the prediction results of Model D were compared with the ASABE drawbar pull equation to examine differences between the empirical formula and the machine learning approach. Third, the models were tested with independent datasets from Seosan and Anseong to verify whether they could operate reliably under soil conditions not represented in the training data, thereby demonstrating their robustness and generalizability.

## Results

### Engine characteristic profile

**Fig. 7:**
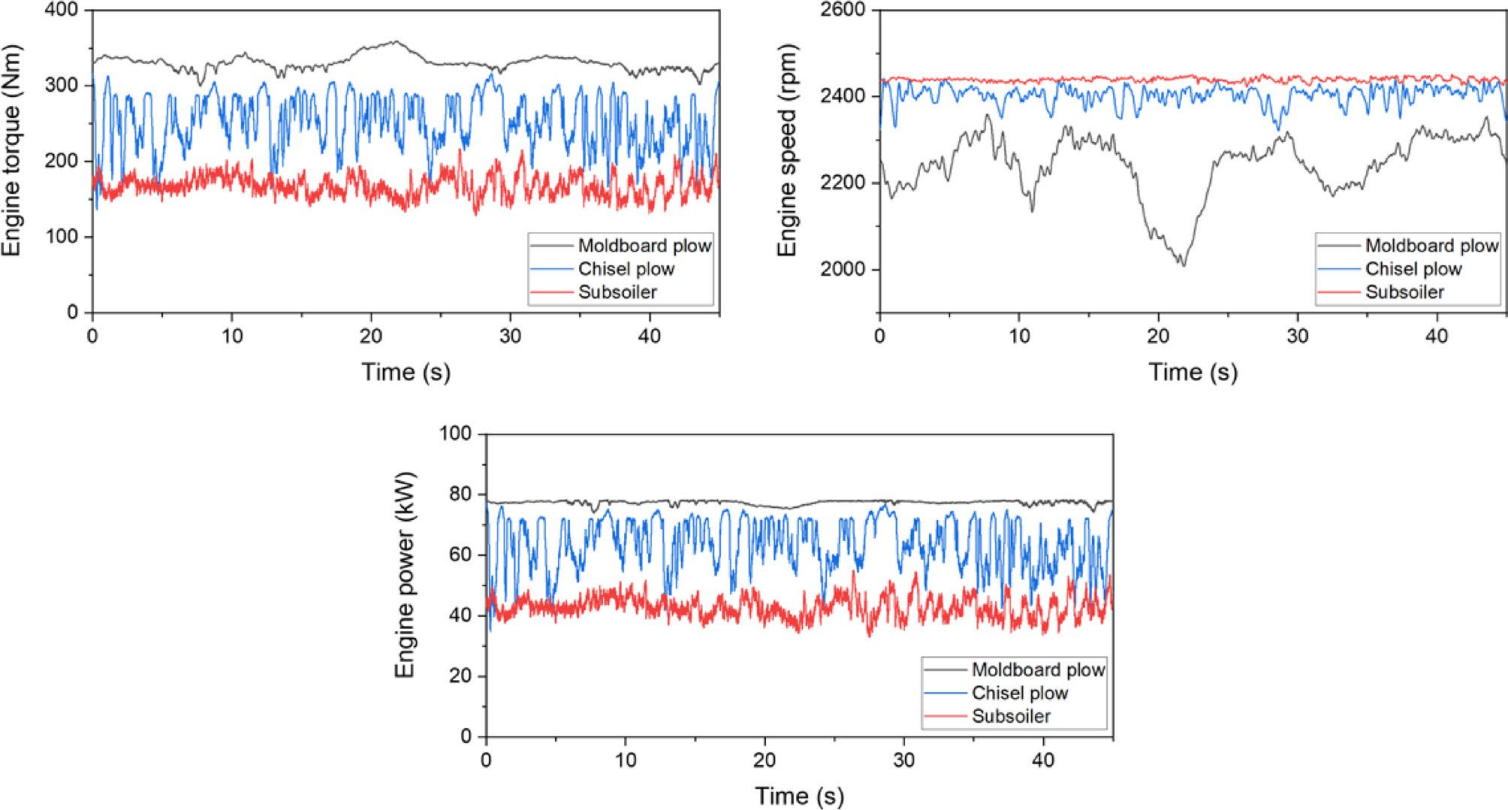
Engine-load characteristics with time for different tillage tools: comparison of engine torque, engine speed, and engine power.

The values of the engine-load measurements are shown in Figure 7. The engine torque and power were the highest for the moldboard plow, followed by the chisel plow and the subsoiler, whereas the engine rotational speed was the highest for the subsoiler, followed by the chisel plow and the moldboard plow. It is believed that, at an equal operating speed, the subsoiler would impose the highest engine load owing to its deeper and wider soil penetration. However, since its operating speed was set lower to avoid excessive draft resistance and slippage, the moldboard plow imposed the highest engine load in the present results.

The results of the statistical analysis of the tractor-engine performance are shown in Table 8. The chisel-plow operation exhibited high CV values for the engine torque and power, indicating significant variations in engine performance during operation. This result suggests that the engine load may fluctuate irregularly, depending on the working conditions or soil resistance. Furthermore, the ANOVA results revealed statistically significant differences in all the engine-performance indicators, clearly demonstrating distinct engine performance characteristics based on the type of plow used.

**Table 8:** Statistical analysis results of engine torque, speed, and power for different types of plows.

Items	Engine torque (Nm)			Engine speed (rpm)			Engine power (kW)		
	M	C	S	M	C	S	M	C	S
Max	359.1	316.9	216.8	2,361	2,446	2,453	78.44	77.08	54.98
Min	299.5	136.8	128.9	2,008	2,322	2,423	73.99	34.96	33.07
Avg	330.1^A^	254.0^B^	167.5^C^	2,243^C^	2,404^B^	2,440^A^	77.44^A^	63.82^B^	42.77^C^
Std	10.1	35.7	14.4	70	22	5	0.76	8.47	3.59
CV	0.031	0.140	0.086	0.031	0.009	0.002	0.010	0.133	0.084

Means with different superscripts (A, B, and C) in each row are significantly different (*p* < 0.05) according to LSD multiple range tests. * M: Moldboard plow; C: Chisel plow; S: Subsoiler.

### Assessment of tractor work performance

**Fig. 8:**
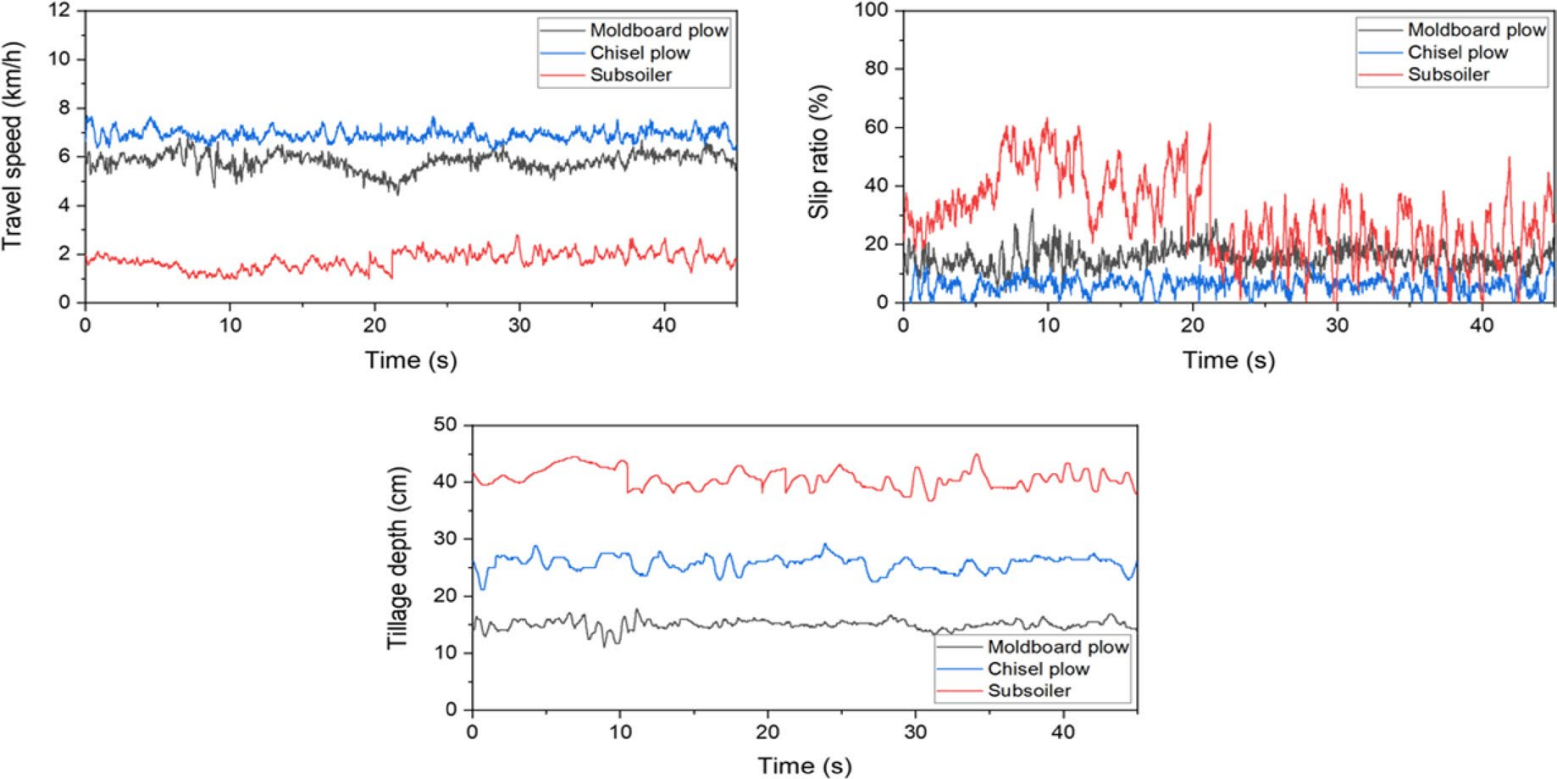
Tractor working-performance metrics with time for different tillage tools: comparison of travel speed, slip ratio, and tillage depth.

The results of the tractor-performance measurements are shown in Figure 8. The subsoiler operation exhibited a high and variable slip ratio, because of the high drawbar resistance relative to the axle power. The travel speed was highest for the chisel plow, followed by the moldboard plow and subsoiler, whereas the slip ratio showed the opposite trend. These differences appear to result from variations in the soil interactions based on the working mechanisms of each plow. The axle power was the highest for the moldboard plow, followed by the chisel plow and subsoiler. These findings indicate that the work performance of a tractor varies depending on the type of plow, highlighting the need for tractor settings that are optimized for specific work characteristics.

The results of the statistical analysis of the tractor performance are presented in Table 9. The CV of the slip ratio was high for both chisel plow and subsoiler operations. Notably, the average slip ratio for the subsoiler is 29.8%, indicating a high level. Additionally, all work-performance metrics exhibited statistically significant differences.

**Table 9:** Statistical analysis results of tractor work performance by plow types.

Items	Travel speed (km/h)			Slip ratio (%)			Tillage depth (cm)		
	M	C	S	M	C	S	M	C	S
Max	6.852	8.112	2.797	32.4	14.6	63.5	17.87	29.25	44.98
Min	4.426	6.278	1.000	3.7	0.0	0.0	11.08	21.20	36.54
Avg	5.795^B^	6.918^A^	1.770^C^	15.8^B^	6.1^C^	29.8^A^	15.03^C^	25.78^B^	40.67^A^
Std	0.357	0.240	0.346	3.9	3.0	13.9	0.89	1.30	1.71
CV	0.062	0.035	0.195	0.245	0.497	0.465	0.059	0.050	0.042

Means with different superscripts (A, B, and C) in each row are significantly different (*p* < 0.05) according to LSD multiple range tests. * M, Moldboard plow; C, Chisel plow; S, Subsoiler.

#### Analysis of traction performance for various tillage implements

**Fig. 9:**
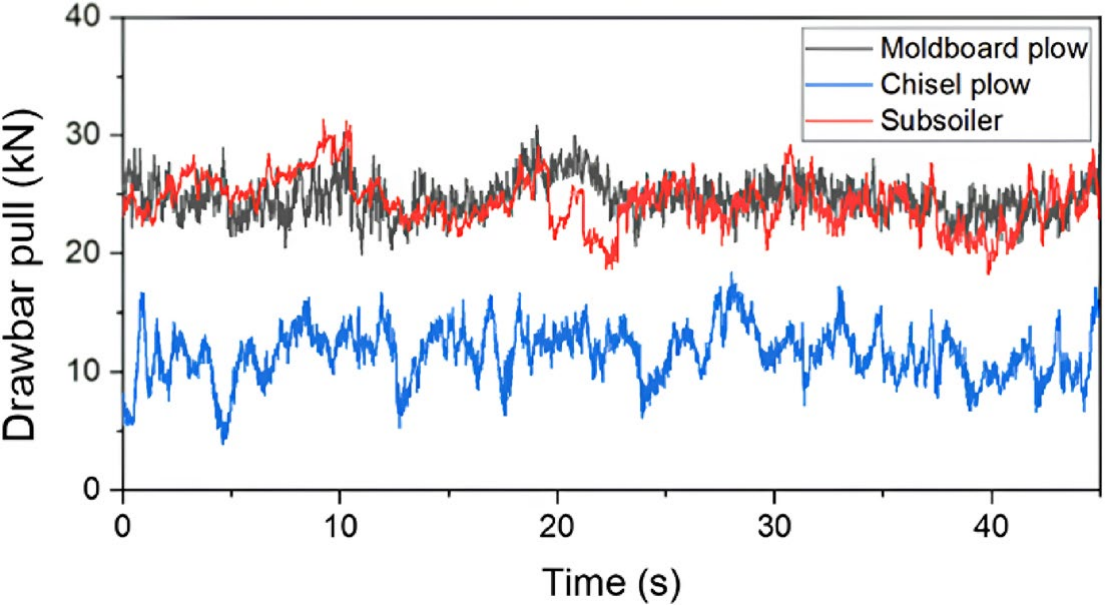
Traction performance factors with time for different tillage tools: comparison of drawbar pull.

The results of the tractor traction performance measurements are shown in Figure 9. High levels of drawbar pull were observed during the moldboard-plow and subsoiler operations, whereas the chisel-plow operation exhibited a highly variable drawbar pull. The drawbar power was the highest for the moldboard plow, followed by the chisel plow and subsoiler, attributed to the lower travel speed of the subsoiler. The traction ratio showed results similar to those of the drawbar pull because no significant differences were observed in the total weight of the tractor. The traction efficiency was highest for the moldboard plow, followed by the chisel plow and subsoiler.

The results of the statistical analysis of the tractor drawbar pull are listed in Table 10. Among all the drawbar pull indicators, the moldboard-plow operation demonstrated the highest drawbar pull. The chisel-plow operation exhibited high CV values across all the drawbar pull indicators. In addition, all drawbar pull metrics showed statistically significant differences.

**Table 10:** Statistical analysis results of drawbar pull by plow types.

Items	Drawbar pull (kN)		
	Moldboard plow	Chisel plow	Subsoiler
Max	30.84	18.41	31.35
Min	19.82	3.88	18.26
Avg	24.73^A^	11.60^C^	24.39^B^
Std	1.61	2.25	2.08
CV	0.065	0.194	0.085

Means with different superscripts (A, B, and C) in each row are significantly different (*p* < 0.05) according to LSD multiple range tests.

**Fig. 10:**
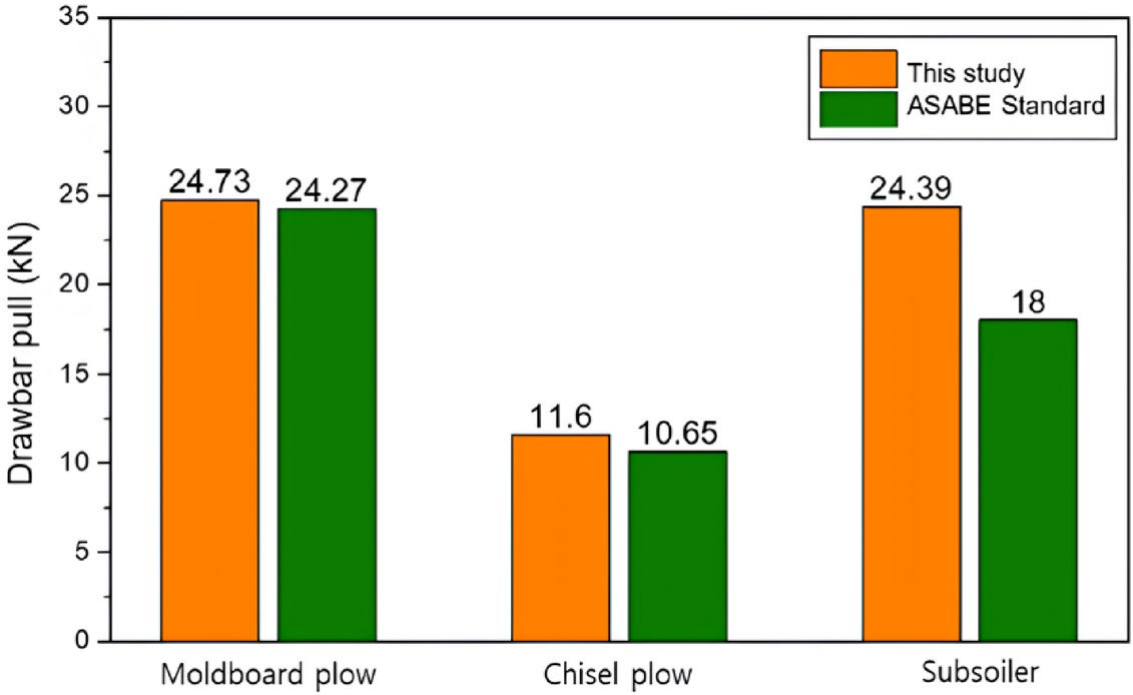
Drawbar pull for different tillage tools: comparison between this study and ASABE standards.

A comparison between the ASAE standard and the measured drawbar pull results is shown in Figure 10. The differences between the moldboard plow, chisel plow, and subsoiler were 1.90, 8.92, and 35.5%, respectively. The larger discrepancy in the subsoiler soil may be attributed to increased slip and other errors caused by deeper tillage. The error margins suggested by the ASAE standards were 40, 50, and 50% for the moldboard plow, chisel plow, and subsoiler, respectively, indicating that the measured data were reliable^20^.

### Results of correlation analysis

**Fig. 11:**
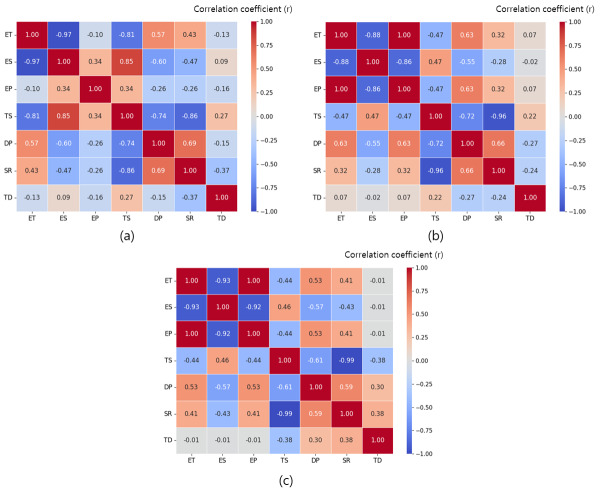
Critical performance data for tractor operations with plow (**a**) moldboard plow, (**b**) chisel plow, and (**c**) subsoiler: Results of correlation analysis.

ET: Engine torque (Nm); ES: Engine speed (rpm); EP: Engine power (kW); TS: Travel speed (km/h); DP: Drawbar pull (kN); SR: Slip ratio (%); TD: Tillage depth (cm).

Figure 11(a) shows the results of the correlation analysis among the major variables during moldboard plow operations. The correlation coefficients (r) of the drawbar pull ranged from -0.74 to 0.69 with key tractor variables. The variables that had the greatest influence on the drawbar pull were travel speed (r = − 0.74) and slip ratio (r = 0.69). Fig. 11(b) shows the results of the correlation analysis among the major variables during chisel plow operations. During chisel plow operations, the correlation coefficients of the drawbar pull ranged from − 0.72 to 0.66 with key tractor variables. The variables that had the greatest influence on the drawbar pull were travel speed (r = − 0.72) and slip ratio (r = 0.66). Fig. 11(c) shows the results of the correlation analysis among the major variables during subsoiler operations. During subsoiler operations, the correlation coefficients of the drawbar pull ranged from − 0.61 to 0.59 with key tractor variables. The variables that had the greatest influence on the drawbar pull were travel speed (r = -0.61), and slip ratio (r = 0.59).

### Machine-learning-based traction-force prediction

**Fig. 12:**
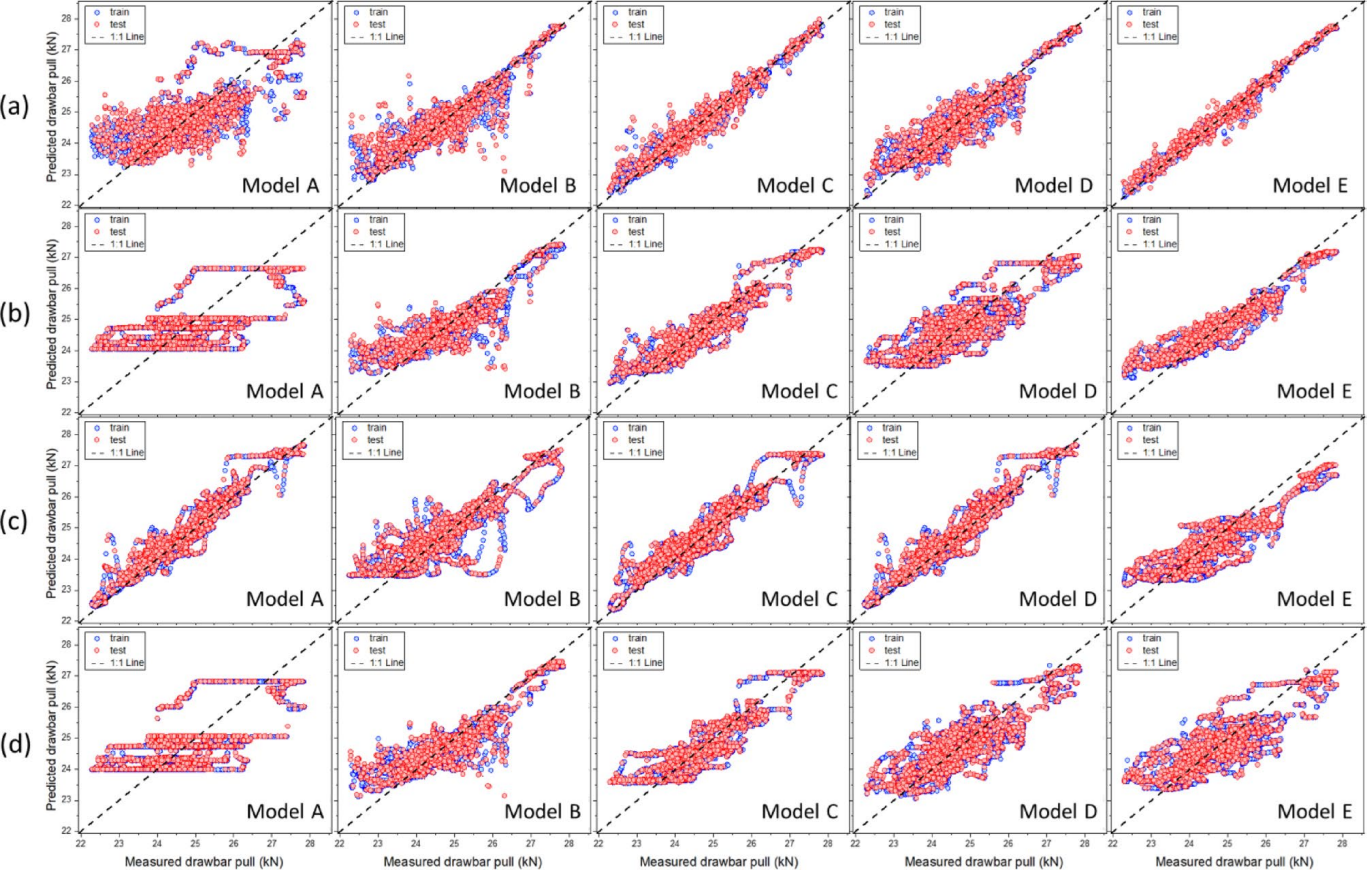
Scatter plots of the measured and predicted drawbar pull for the moldboard plow: (a) RF, (b) XGB, (c) ANN, and (d) SVM.

*Moldboard plow* Figure 12 shows the results of the traction-force prediction model for the moldboard plow, comparing the measured and predicted drawbar pulls across the five variable combinations. The model development results indicated that, except for Model A of the XGB and SVM, the predicted drawbar pull was closely aligned with the measured drawbar pull along the 1:1 reference line. Table 11 presents the performance metrics of the various machine-learning algorithms, including RF, XGB, ANN, and SVM. These algorithms were used to predict the drawbar pulls of a moldboard plow. The model performance was evaluated using the R^2^, RMSE, MAPE, and RD metrics for both the training and test datasets. Additionally, model overfitting was assessed based on R^2^ Gap and TTLR values. Among the five models, the performance of Model A was lower than that of the other four models. However, except for Model A, the R^2^ values of the remaining four models exceeded 0.68, with MAPE within 2.6%, RMSE below 0.81 kN, and RD under 3.21%, demonstrating stable predictive performance. Model E, which employed RF, achieved the highest predictive accuracy, with an R^2^ value of 0.977, RMSE of 0.118 kN, MAPE of 0.44%, and RD of 0.47%. The overfitting-assessment results show that the TTLR values for the RF model range from 1.266–1.856, indicating a slight risk of overfitting. However, as the R^2^ Gap values remained within an acceptable range (0.013–0.138), the generalization performance of the model can be considered reliable.

**Table 11:** Performance evaluation of machine-learning models for predicting drawbar pull in moldboard-plow operations.

Model		R^2^			RMSE (kN)			MAPE (%)		RD (%)	
		Train	Test	R^2^ Gap	Train	Test	Test	Train	Test	Train	Test
A	RF	0.633	0.495	0.138	0.645	0.746	1.337	1.82	2.14	2.61	3.02
	XGB	0.460	0.429	0.031	0.783	0.794	1.028	2.44	2.53	3.17	3.21
	ANN	0.618	0.601	0.016	0.305	0.330	1.166	0.91	0.97	0.01	0.01
	SVM	0.482	0.390	0.092	0.772	0.809	1.099	0.02	0.03	0.03	0.03
B	RF	0.889	0.793	0.096	0.354	0.483	1.856	0.89	1.17	0.01	1.95
	XGB	0.801	0.731	0.071	0.479	0.536	1.255	1.39	1.48	1.94	2.17
	ANN	0.782	0.753	0.030	0.497	0.523	1.104	1.40	1.45	0.02	0.02
	SVM	0.816	0.770	0.046	0.452	0.516	1.305	1.29	1.43	1.83	2.09
C	RF	0.968	0.944	0.024	0.190	0.227	1.432	0.52	0.69	0.01	0.92
	XGB	0.898	0.877	0.020	0.338	0.376	1.237	0.01	0.01	0.01	0.02
	ANN	0.902	0.887	0.015	0.333	0.353	1.124	1.04	1.10	0.01	0.01
	SVM	0.815	0.800	0.015	0.459	0.469	1.044	0.01	0.02	0.02	0.02
D	RF	0.879	0.846	0.032	0.368	0.420	1.299	1.06	1.20	0.01	1.70
	XGB	0.704	0.718	− 0.014	0.574	0.572	0.991	1.84	1.83	2.32	2.31
	ANN	0.918	0.901	0.016	0.305	0.330	1.166	0.91	0.97	0.01	0.01
	SVM	0.737	0.741	− 0.004	0.538	0.556	1.071	1.71	1.75	2.17	2.25
E	RF	0.990	0.977	0.013	0.105	0.118	1.266	0.28	0.44	0.00	0.48
	XGB	0.881	0.858	0.023	0.368	0.395	1.157	1.13	1.20	1.49	1.60
	ANN	0.772	0.754	0.018	0.509	0.522	1.049	1.61	1.65	0.02	0.02
	SVM	0.684	0.699	− 0.016	0.586	0.607	1.072	1.82	1.89	2.37	2.45

**Fig. 13:**
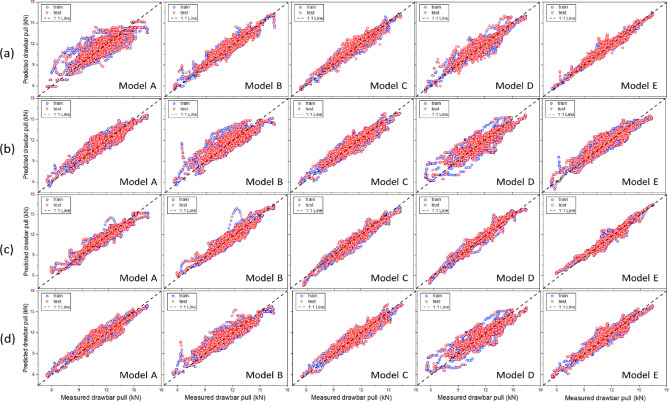
Scatter plots of the measured and predicted drawbar pull for the chisel plow: (**a**) RF, (**b**) XGB, (**c**) ANN, and (**d**) SVM.

*Chisel plow* Fig. 13 shows the results of the traction-force prediction model for the chisel plow, comparing the measured and predicted drawbar pulls across the five variable combinations. The Model A development results indicated that, except for RF Model A, the predicted drawbar pull was closely aligned with the measured drawbar pull along the 1:1 reference line. Additionally, the agreement with the 1:1 reference line improved as the number of variables increased. Table 12 summarizes the performance metrics of the chisel plow. The analysis results indicated that all models achieved an R^2^ value of at least 0.73. Model A, which used only the tractor-engine variables (engine torque and engine speed) as input variables, had an average R^2^ value of 0.860. In contrast, Model B, which incorporated the tillage depth in addition to the tractor engine variables (engine torque and engine speed), showed an improved average R^2^ value of 0.874. Furthermore, Model C, which included travel speed along with the tractor-engine variables (engine torque and engine speed), achieved the highest predictive performance with an average R^2^ value of 0.911. These findings confirm that including additional variables, such as tillage depth or travel speed, improves the model’s predictive performance compared to using only tractor-engine variables. The overfitting-assessment results showed that the R^2^ gap remained below 0.049 and the TTLR remained under 1.429, demonstrating an overall appropriate level of model generalization.

**Table 12:** Performance evaluation of machine learning models for predicting drawbar pull in chisel-plow operations.

Model		R^2^			RMSE (kN)			MAPE (%)		RD (%)	
		Train	Test	R^2^ Gap	Train	Test	TTLR	Train	Test	Train	Test
A	RF	0.756	0.733	0.023	0.908	0.951	1.098	6.04	6.26	7.79	8.16
	XGB	0.897	0.884	0.012	0.592	0.626	1.121	4.13	4.37	5.08	5.37
	ANN	0.923	0.916	0.007	0.510	0.535	1.099	3.32	3.56	0.04	0.05
	SVM	0.923	0.908	0.015	0.511	0.559	1.199	0.03	0.04	0.04	0.05
B	RF	0.936	0.912	0.023	0.466	0.547	1.379	3.16	3.70	4.00	4.69
	XGB	0.800	0.786	0.014	0.818	0.863	1.112	5.58	6.00	7.02	7.40
	ANN	0.959	0.953	0.006	0.215	0.227	1.116	0.61	0.64	0.02	0.02
	SVM	0.875	0.846	0.029	0.647	0.732	1.281	4.49	5.04	5.55	6.28
C	RF	0.958	0.929	0.029	0.373	0.445	1.429	2.43	3.26	3.20	3.82
	XGB	0.915	0.900	0.015	0.536	0.580	1.170	0.04	0.04	0.05	0.05
	ANN	0.918	0.901	0.016	0.305	0.330	1.166	0.91	0.97	0.03	0.03
	SVM	0.938	0.913	0.025	0.457	0.541	1.401	0.03	0.04	0.04	0.05
D	RF	0.911	0.861	0.049	0.652	0.684	1.102	3.54	4.51	5.59	5.87
	XGB	0.810	0.805	0.006	0.802	0.810	1.020	5.56	5.54	6.88	6.95
	ANN	0.959	0.950	0.009	0.373	0.412	1.223	2.40	2.64	0.03	0.04
	SVM	0.809	0.805	0.005	0.805	0.810	1.015	5.60	5.57	6.90	6.95
E	RF	0.969	0.947	0.023	0.374	0.424	1.285	2.06	2.68	3.21	3.64
	XGB	0.873	0.876	− 0.003	0.655	0.652	0.990	4.49	4.54	5.62	5.59
	ANN	0.772	0.754	0.018	0.509	0.522	1.049	1.61	1.65	0.04	0.04
	SVM	0.925	0.920	0.005	0.503	0.522	1.075	3.39	3.49	4.32	4.48

**Fig. 14:**
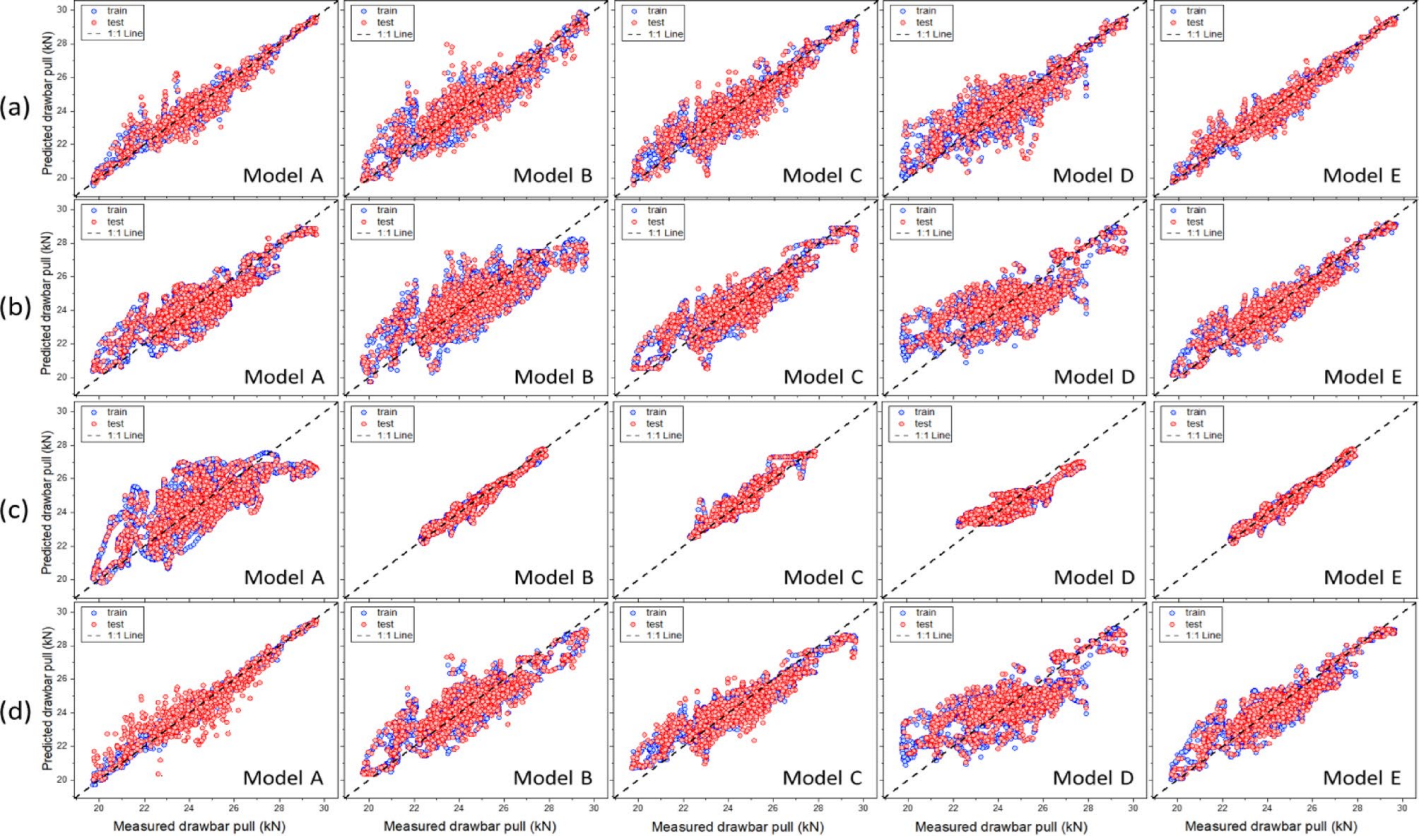
Scatter plots of the measured and predicted drawbar pull for the subsoiler: (**a**) RF, (**b**) XGB, (**c**) ANN, and (**d**) SVM.

*Subsoiler* Fig. 14 shows the results of the traction-force prediction model for the subsoiler, comparing the measured and predicted drawbar pulls across the five variable combinations. The model development results indicated that in all cases, the predicted drawbar pull was closely aligned with the measured drawbar pull along the 1:1 reference line. Model E, which included engine performance, draft depth, and speed sensor data, exhibited the highest agreement with the 1:1 reference line. Table 13 summarizes the performance metrics of the subsoiler. The analysis results indicated that Model D, which used tillage depth and travel speed as input variables while excluding the tractor-engine variables, had the lowest average R^2^ value of 0.671. By contrast, Model E, which incorporated tractor-engine variables along with tillage depth and travel speed, achieved the highest average R^2^ value of 0.908. These findings suggest that an appropriate combination of engine- and tractor-related variables is necessary to optimize the performance of the traction-force prediction model. The overfitting-assessment results showed that Model A of the SVM exhibited a high TTLR value of 8.506, suggesting a potential overfitting problem in the model with respect to the training data. However, owing to the nature of SVM, the TTLR values can be relatively high. Considering SVM operates by separating data in a high-dimensional space, it may react sensitively to training data, leading to an increase in TTLR values for certain datasets.

**Table 13:** Performance evaluation of machine learning models for predicting drawbar pull in subsoiler operations.

Model		R^2^			RMSE (kN)			MAPE (%)		RD (%)	
		Train	Test	R^2^ Gap	Train	Test	TTLR	Train	Test	Train	Test
A	RF	0.967	0.916	0.051	0.446	0.538	1.452	0.93	1.40	3.83	2.20
	XGB	0.822	0.783	0.039	0.796	0.863	1.175	2.59	2.83	3.26	3.54
	ANN	0.560	0.552	0.008	1.253	1.240	0.979	3.95	3.56	0.05	0.05
	SVM	0.987	0.881	0.105	0.219	0.638	8.506	0.01	0.02	0.01	0.03
B	RF	0.876	0.799	0.077	0.711	0.848	1.423	1.98	2.55	6.10	3.48
	XGB	0.698	0.663	0.036	1.028	1.098	1.139	3.35	3.55	4.22	4.50
	ANN	0.959	0.953	0.006	0.215	0.227	1.116	0.61	0.64	0.01	0.01
	SVM	0.818	0.768	0.050	0.799	0.910	1.299	2.55	2.90	3.27	3.73
C	RF	0.902	0.827	0.075	0.638	0.785	1.516	1.74	2.40	5.47	3.22
	XGB	0.839	0.791	0.047	0.752	0.862	1.313	0.02	0.03	0.03	0.04
	ANN	0.918	0.901	0.016	0.305	0.330	1.166	0.91	0.97	0.01	0.01
	SVM	0.888	0.817	0.071	0.628	0.809	1.657	0.02	0.03	0.03	0.03
D	RF	0.845	0.749	0.096	0.737	0.948	1.654	2.22	2.79	6.32	3.88
	XGB	0.618	0.590	0.028	1.157	1.211	1.096	3.89	4.10	4.74	4.96
	ANN	0.772	0.754	0.018	0.509	0.522	1.049	1.61	1.65	0.02	0.02
	SVM	0.614	0.592	0.022	1.163	1.209	1.080	3.94	4.12	4.77	4.96
E	RF	0.966	0.934	0.032	0.395	0.479	1.469	0.95	1.37	3.39	1.96
	XGB	0.912	0.888	0.024	0.559	0.627	1.258	1.76	1.97	2.29	2.57
	ANN	0.959	0.953	0.006	0.215	0.227	1.116	0.61	0.64	0.01	0.01
	SVM	0.867	0.855	0.012	0.685	0.712	1.080	2.17	2.25	2.81	2.92

### Comparison of the ASABE traction equation and machine learning based prediction models

Table 14 presents the results of comparing the performance of the ASABE drawbar pull equation and machine learning models across different plow types. Among the machine learning models, Model D was constructed using the same input variables as the ASABE equation, namely tillage depth and travel speed. Based on the results, the ASABE drawbar pull equation showed very low coefficients of determination (R^2^ = 0.029–0.158) across all plow types, indicating its limited ability to explain variations in drawbar pull. According to ASABE Standard (D497.4), an error of approximately ±40% has been reported for moldboard plows, and about ±50% for chisel plows and subsoilers^20^. However, in this study the equation performed even worse than the error ranges reported in the ASABE standard. One possible explanation is that the empirical formula was developed using data that are thought to reflect soil and tillage conditions in the United States, while the datasets applied here came from Korean fields with different soil properties and management practices. These regional differences may have widened the gap between the actual drawbar pull observed in the experiments and the simplified predictors included in the ASABE equation. Moreover, this is considered to be due to the fact that it does not account for key powertrain variables such as engine torque, engine speed, and slip ratio^36^. Instead, it is considered to primarily rely on implement-related factors such as working speed, implement width, and tillage depth. In contrast, the machine learning models achieved substantially higher R^2^ values, with ANN exhibiting the best performance for the chisel (0.950) and moldboard (0.901) plows. For the subsoiler, RF (0.749) and ANN (0.754) outperformed the other models.

**Table 14:** Comparison R^2^ of ASABE drawbar pull equation and machine learning models across different plow types.

Plow type	ASABE Standard	Machine learning (Model D)			
		RF	XGB	ANN	SVM
Moldboard	0.158	0.846	0.718	0.901	0.741
Chisel	0.155	0.861	0.805	0.950	0.805
Subsoiler	0.029	0.749	0.590	0.754	0.592

### Comparison and evaluation of machine-learning-based predictive models

**Fig. 15:**
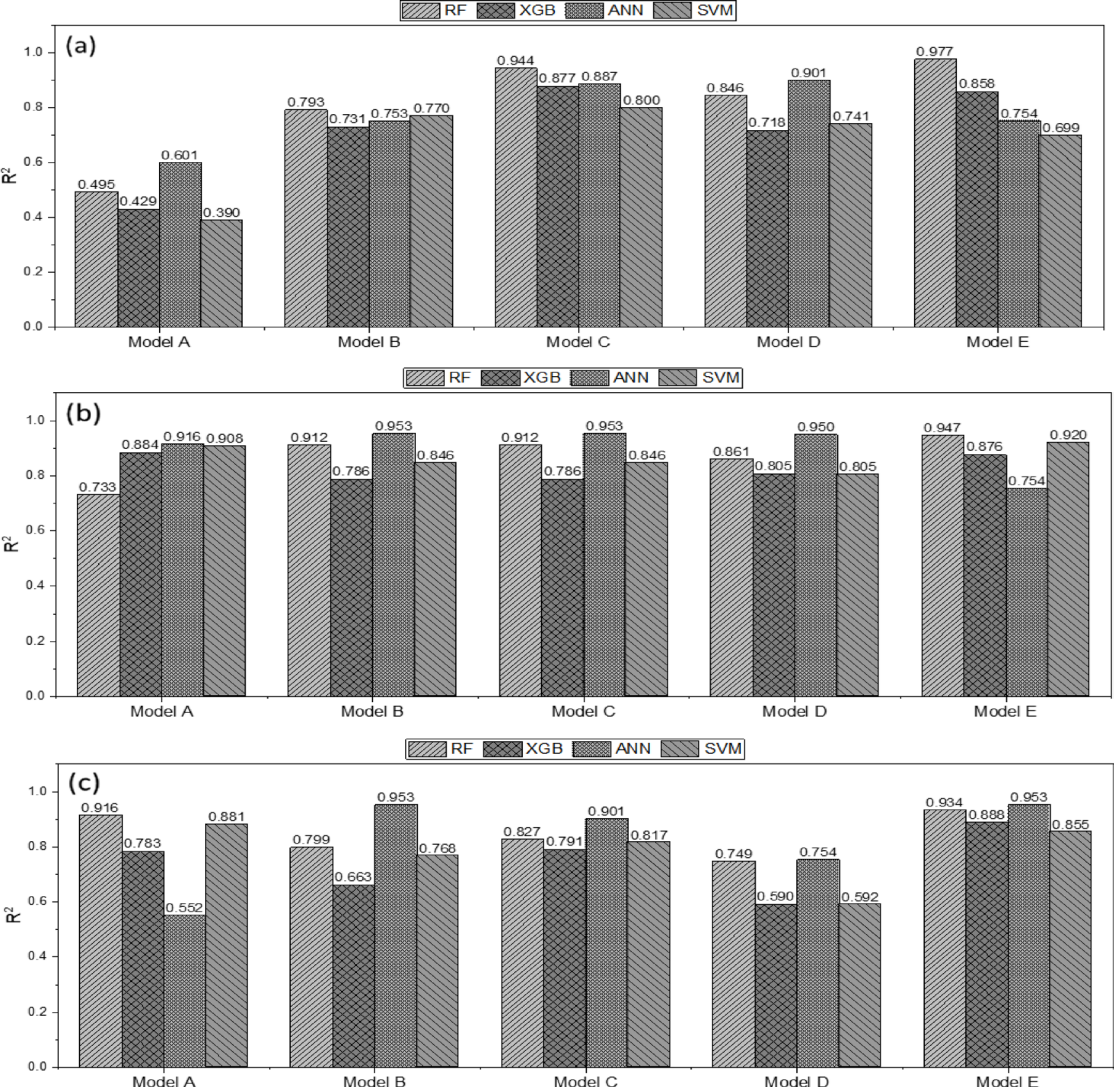
Traction-force prediction for different tillage tools: (**a**) moldboard plow, (**b**) chisel plow, and (**c**) subsoiler. Comparison of R^2^ among machine-learning Models (**A–E**): Model A = drawbar pull as a function of engine speed and engine torque; Model B = drawbar pull as a function of engine speed, engine torque, and tillage depth; Model C = drawbar pull as a function of engine speed, engine torque, travel speed, and slip ratio; Model D = drawbar pull as a function of tillage depth and travel speed; Model E = drawbar pull as a function of engine speed, tillage depth, and travel speed.

Figure 15 shows the traction-force prediction performance of the machine-learning models, presenting the R^2^ values for each type of plow. For the moldboard plow, Model A exhibited the lowest R^2^ values, with XGB and SVM showing the lowest performances of 0.429 and 0.390, respectively. In Model C, the R^2^ values improved significantly across all models, with RF and ANN achieving high performances of 0.944 and 0.887, respectively. Overall, Models C and E demonstrated superior predictive performances, with RF showing the highest accuracy. For the chisel plow, Model A showed relatively high R^2^ values, and all models maintained a certain level of predictive performance. The R^2^ values ranged from 0.733–0.953, with RF and XGB achieving the highest predictive accuracies. For the subsoiler, the ANN exhibited the highest predictive performance in Models B and E, with R^2^ values of 0.953. RF consistently maintained high performance across all models, particularly in Models A (0.916) and E (0.934). In contrast, SVM and XGB had relatively lower predictive performances than RF and ANN.

**Fig. 16:**
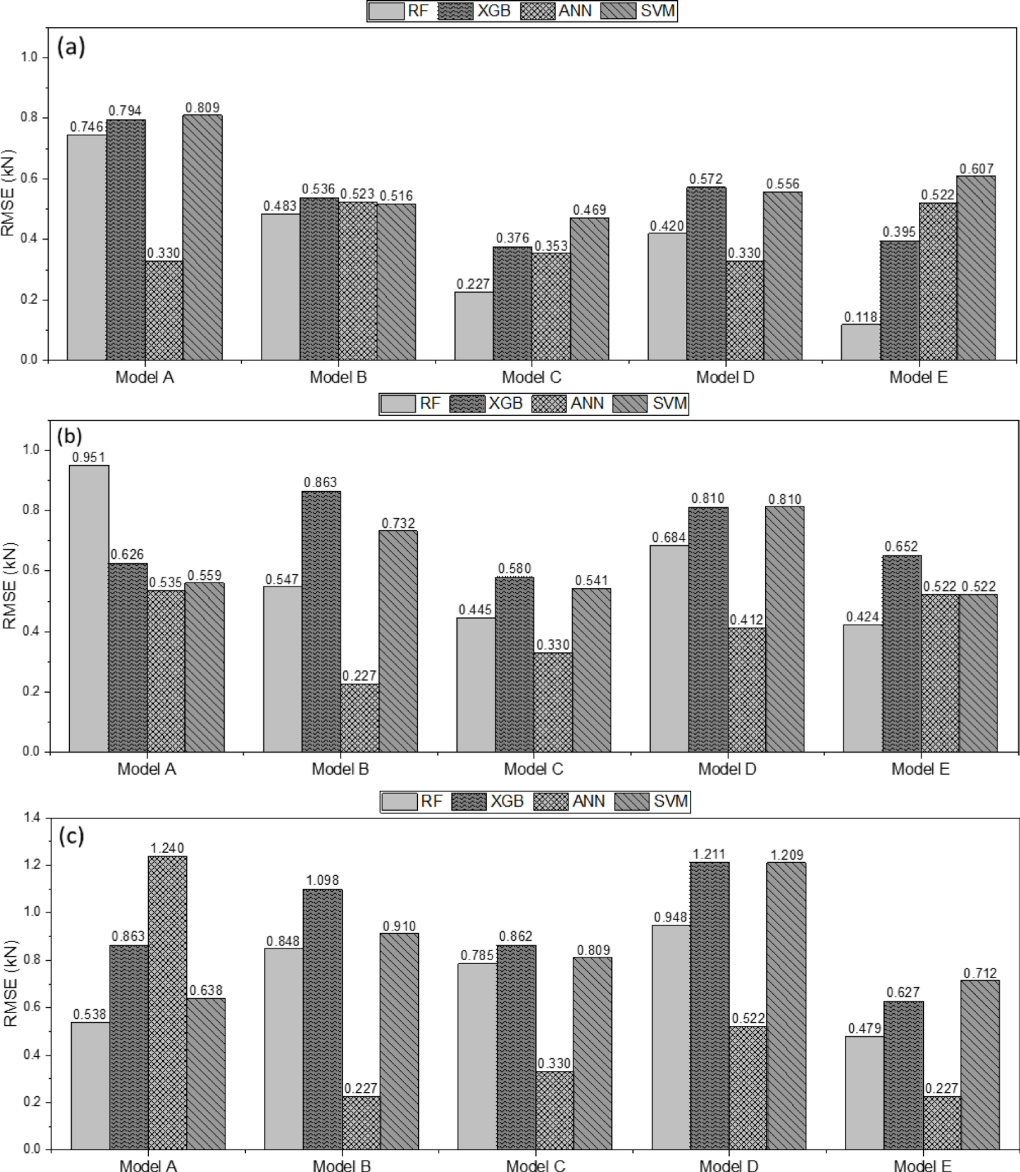
Traction-force prediction for different tillage tools: (**a**) moldboard plow, (**b**) chisel plow, and (**c**) subsoiler. Comparison of RMSE among machine-learning Models (**A–E**): Model A = drawbar pull as a function of engine speed and engine torque; Model B = drawbar pull as a function of engine speed, engine torque, and tillage depth; Model C = drawbar pull as a function of engine speed, engine torque, travel speed, and slip ratio; Model D = drawbar pull as a function of tillage depth and travel speed; Model E = drawbar pull as a function of engine speed, tillage depth, and travel speed.

Figure 16 presents the RMSE values for each type of plow, comparing the traction-force prediction performance of the machine-learning models. Across all plow types, the ANN and RF recorded the lowest RMSE values. Additionally, Model E, which incorporated tractor-engine variables (engine speed) along with tillage depth and travel speed, achieved lower RMSE values than Model A, which only used the tractor-engine variables (engine torque and engine speed).

### Results of robustness test

**Fig. 17:**
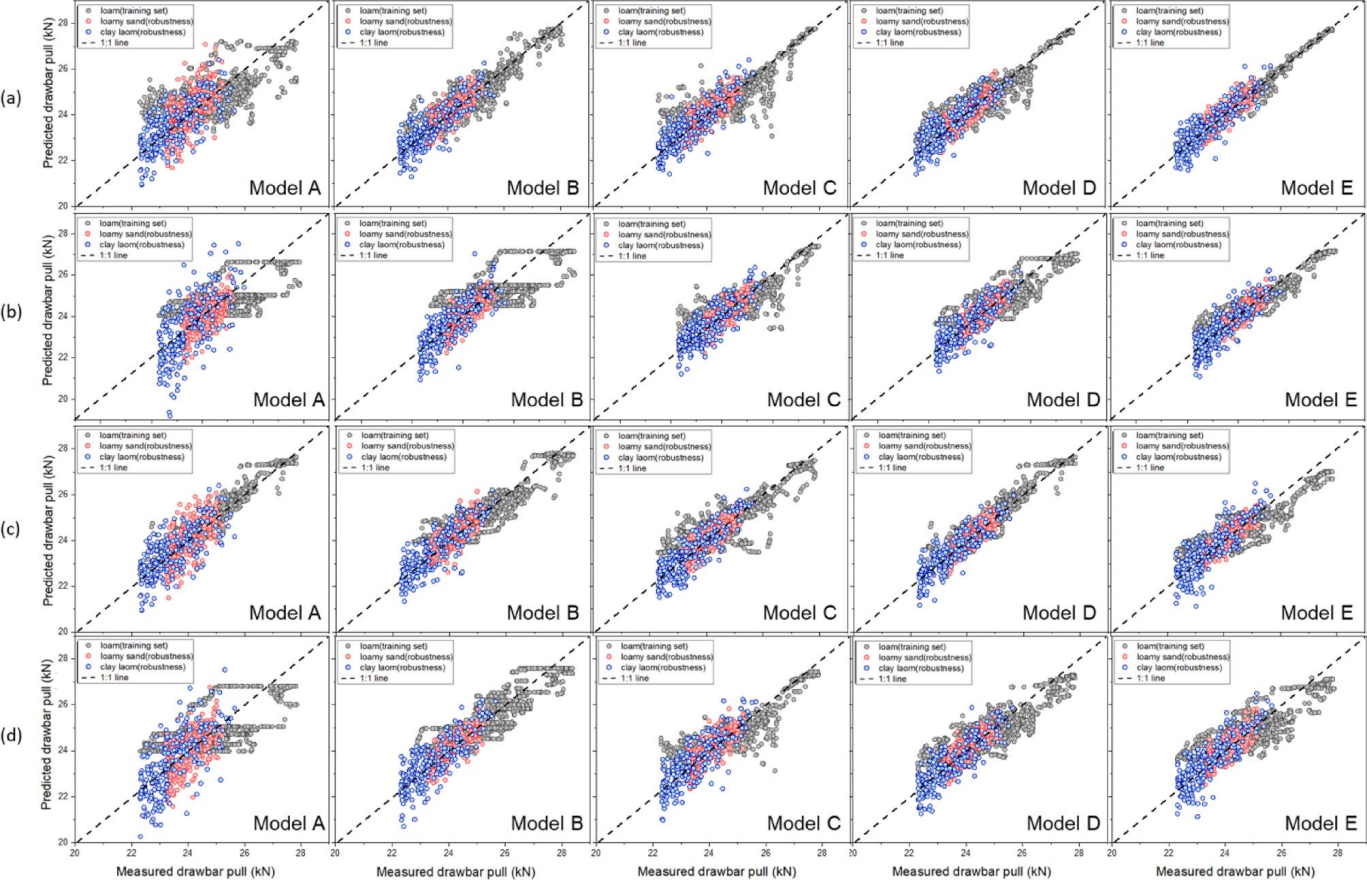
Robustness test using scatter plots of the measured and predicted values obtained with the moldboard plow: (**a**) RF, (**b**) XGB, (**c**) ANN, and (**d**) SVM.

Figure 17 presents the results of the robustness test based on scatter plots of the measured and predicted values obtained using the moldboard plow. In the model development process, the R^2^ values of the test set ranged from 0.390 to 0.977, with values of 0.390 to 0.777 in loamy sand soils and 0.405 to 0.775 in clay loam soils. These results suggest that the model, which had been trained on loam soils, still produced meaningful predictions when applied to other soil types, although its accuracy declined. The lower R^2^ values observed during external validation can be explained by the strong effect of soil physical properties on drawbar pull. Earlier studies have reported that, in robustness checks using external datasets, R^2^ close to 0.7 are often considered an acceptable result^37^. These results show that the model keeps a practical level of robustness with diverse soil data. At the same time, adding training data from a wider range of soils will be important for improving the model’s generalizability.

## Discussion

In this study, models for predicting the tractor drawbar pull were developed based on RF, XGB, ANN, and SVM. The performance of these models was compared across five variable combinations. The analysis of the results showed that the traction-force predictions were dependent on the type of plow and combination of input variables. As the number of input variables increased, model performance improved; incorporating tillage depth and travel speed in addition to tractor-engine parameters led to improved prediction accuracy.

For the moldboard plow, the prediction performance of Model A, which applied XGB and SVM, was relatively low, with R^2^ values of 0.429 and 0.390, respectively, indicating a lower accuracy than the other models. However, in Model C, the prediction performances of all models significantly improved, with RF and ANN achieving R^2^ values of 0.944 and 0.887, respectively, demonstrating superior performance. These results suggest that using engine speed, engine torque, travel speed, and slip ratio as input variables and applying the RF are the most effective approaches for predicting the action force of a moldboard plow. For the chisel plow, all the models maintained R^2^ values above 0.733, indicating a relatively stable performance. RF and XGB demonstrate the highest prediction accuracies. However, for the subsoiler, the ANN achieved the highest performance in Models B and E, with R^2^ = 0.953, whereas the RF model also achieved the same R^2^ = 0.953 in Model E, demonstrating stable performance. These results indicate that when predicting the action force of a subsoiler, setting engine speed, tillage depth, and travel speed as input variables is advantageous for maximizing model performance.

Overall, the RF model achieved a superior performance across all three plow types. However, the TTLR value for the RF model ranges from 1.266–1.856, suggesting a potential risk of overfitting. This result indicates that while the RF-based model performed well on the training data, additional adjustments are necessary to ensure the generalization of the test data.

The robustness test results showed that the R^2^ values ranged from 0.390 to 0.777 for loamy sand and from 0.405 to 0.775 for clay loam, which were lower than the performance metrics obtained during model development. Nevertheless, these values can be considered reasonable compared to previous studies. However, further research is needed to incorporate more diverse soil conditions and improve the generalization performance of the model.

## Conclusions

This paper presents a machine-learning-based approach for estimating tractor drawbar pull, providing a cost-effective alternative to expensive load cells. The primary objective of this study was to identify the optimal machine learning technique and input variable combinations for each plow type (moldboard plow, chisel plow, and subsoiler). The proposed methodology can enhance precision agriculture by enabling real-time performance monitoring and optimization.

We developed a tractor traction-force prediction model using four machine-learning techniques, incorporating key tractor parameters as input variables. The R^2^ values for each model (RF, XGB, ANN, and SVM) were 0.495, 0.977, 0.429, 0.888, 0.552, 0.953, and 0.390, 0.920, respectively. However, the model performance varies significantly depending on the type of plow and input variables used. A comparison of the four models indicates that increasing the number of input variables is a key factor in improving the R2 values. Models incorporating travel speed, tillage depth, and slip ratio, in addition to engine data, exhibited superior performance compared to those using only engine parameters.

However, this study has several limitations. First, the data were collected under restricted conditions in specific agricultural fields, which limits the generalizability of the findings, particularly due to the failure to account for diverse soil conditions such as sandy or clay soils. Secondly, the dataset was not sufficiently large, necessitating additional data collection for a more reliable analysis. Third, various influencing factors were not considered simultaneously as the analysis was conducted on individual variables.

These limitations will be addressed in future studies using field experiments to collect data under diverse working conditions. Future research should focus on improving model reliability by applying hyperparameter optimization and regularization techniques to mitigate overfitting.

Finally, this paper proposes a methodology for applying various machine-learning techniques to tractor traction-force prediction. This study demonstrated that machine learning techniques utilizing relatively low-cost sensors can serve as effective alternatives to conventional complex methods for measuring drawbar pulls. With the advancements in agricultural technology, the models developed in this study are expected to have various practical applications.

## Data Availability

The datasets used and analyzed during the current study available from the corresponding author on reasonable request.
